# Resources on Alcohol-Related Birth Defects

**Published:** 2001

A number of important associations and organizations have been developed to educate, inform, and assist clinicians and the public about the consequences of prenatal alcohol exposure. The following list, though not exhaustive, is designed to give readers an idea of some of the resources that exist on the topic of alcohol-related birth defects.

## The Arc of the United States

National Headquarters Office
  1010 Wayne Avenue
  Suite 650
  Silver Spring, MD 20910
  Phone: (301) 565-3842
  Fax: (301) 565-5342
  Info@TheArc.org
  http://www.thearc.org

The Arc is the national organization of and for people with mental retardation and related developmental disabilities and their families. Through education, research, and advocacy, The Arc works to improve the quality of life for children and adults with mental retardation and related developmental disabilities and their families and works to prevent both the causes and the effects of mental retardation. The Arc’s FAS Resource and Materials Guide and a comprehensive list of FAS information and related links can be found at the website listed above.

## Consortium for Citizens with Disabilities

1730 K Street, N.W., Suite 1212
  Washington, DC 20006
  Phone: (202) 785-3388
  Fax: (202) 467-4179
  Info@c-c-d.org
  http://www.c-c-d.org/index.htm

The Consortium for Citizens with Disabilities is a coalition of national disability organizations working together to advocate for national public policy that ensures the self-determination, independence, empowerment, integration and inclusion of children and adults with disabilities in all aspects of society.

## Family Empowerment Network: Supporting Families Affected by FAS/FAE (FEN)

Department of Professional Development and Applied Studies
  University of Wisconsin-Madison
  610 Langdon Street
  Room 519
  Madison, WI 53703-1195
  Phone: (800) 462-5254
  Fax: (608) 265-3352
  fen@dcs.wisc.edu
  http://www.dcs.wisc.edu/pda/hhi/fen

FEN is a national resource, referral, and support organization serving families affected by FAS and FAE and the providers who work with them. FEN is a project of the University of Wisconsin-Madison, Division of Continuing Studies, Department of Professional Development and Applied Studies. In addition to providing information and support, FEN also helps families by organizing family retreats, educational and training programs, a national conference, and by publishing a newsletter and providing other information and materials.

## FAS World

FAS World Toronto
  250 Scarborough Golf Club Road
  Toronto, Ontario
  Canada
  M1J 3G8
  Phone: (416) 264-8000
  Fax: (416) 264-8222
  fasworldcanada@home.com

The mission of FAS World is to raise awareness about the effects of prenatal alcohol exposure. The organization aims to educate leaders, lawmakers, and other policymakers around the world so that they can better make decisions and formulate laws and policies that will ensure a healthier society by reducing the incidence of FAS and other alcohol-related disabilities and by providing needed intervention services to those already affected.

## FAS/FAE Information Service

Canadian Centre on Substance Abuse
  75 Albert Street, Suite 300
  Ottawa, Ontario
  Canada
  K1P 5E7
  Phone: (800) 559-4514, (613) 235-4048
  Fax: (613) 235-8101
  fas@ccsa.ca
  http://www.ccsa.ca/fasgen.htm

This information service, operated by the Canadian Centre on Substance Abuse, provides links to support groups, prevention projects, resource centers, and experts on FAS/FAE. It also provides information on FAS/FAE to a variety of clients, including caregivers, educators, social workers, health care and treatment professionals, members of the legal community, policymakers and planners, researchers, and the general public.

## Fetal Alcohol and Drug Unit

Department of Psychiatry and Behavioral Sciences
  University of Washington School of Medicine
  180 Nickerson Street, Suite 309
  Seattle, WA 98109-9112
  Phone: (206) 543-7155
  Fax: (206) 685-2903
  http://depts.washington.edu/fadu

The Fetal Alcohol and Drug Unit conducts research relating to the prevention, intervention, and treatment of FAS and FAE. The unit also disseminates information on fetal alcohol and drug effects and provides consultation for those affected by prenatal exposure to alcohol.

## Fetal Alcohol Education Program

1975 Main Street
  Concord, MA 01742
  Phone: (978) 369-7713
  Fax: (978) 287-4993

The Fetal Alcohol Education Program (FAEP) is dedicated to research and education for the prevention, identification, and treatment of alcohol-related neurodevelopmental disorders. The program has a number of educational materials available for sale, including two teaching packages, one for professional education and one for parents and community groups. They also have developed a handbook for parents. In addition, FAEP staff offers brief consultations and referrals and maintains a list of families interested in participating in research studies.

## Fetal Alcohol Syndrome Consultation, Education and Training Services, Inc (FASCETS): Providing Alternative Paradigms for Understanding Facets of Behavior

P.O. Box 83175
  Portland, OR 97283
  Phone/fax: (503) 621-1271
  dmalbin@fascets.org
  http://www.fascets.org

FASCETS is a private, nonprofit organization for parents and professionals that offers services designed to increase understanding, build on strengths, expand options for developing effective parenting and professional techniques, enhance existing programs, and support the development of new programs. FASCETS offers community education through conferences, workshops, and professional seminars and provides professional development and consultation services.

## Fetal Alcohol Syndrome Family Resource Institute (FASFRI)

P.O. Box 2525
  Lynnwood, WA 98036
  Phone: (253) 531-2878
  vicfas@hotmail.com
  http://www.fetalalcoholsyndrome.org

FASFRI is a nonprofit organization that provides a variety of resources and services to families affected by FAS and ARBD. The Institute’s services include referrals and support in finding help, parent support and advocacy groups, information for parents and professionals, a newsletter and brochures, prevention programs, as well as training sessions and workshops.

## Healthy Start Hotline

Phone: (800) 311-BABY (English)
  Phone: (800) 504-7081 (Spanish)

By calling this hotline, women can reach either their own State’s maternal and child health program or a local Healthy Start site, whichever is closer. The National Hispanic Prenatal Hotline provides answers to questions about prenatal issues in English and Spanish, mails out culturally appropriate information, and acts as a resource to providers who work with Hispanic families on prenatal care issues. Callers are told which prenatal care services are located nearest to where they live. Hotlines also put people in touch with other services; organizations; and agencies, including assistance programs, prenatal clinical care, home visiting services, translation services, parenting classes, and substance abuse treatment.

## Iceberg

Fetal Alcohol Syndrome Information Services (FASIS)
  P.O. Box 95597
  Seattle, WA 98145-2597
  Phone: (425) 827-1773
  iceberg_fas@yahoo.com

Iceberg is an educational newsletter for people concerned about FAS and FAE.

## March of Dimes Birth Defects Foundation Resource Center

1275 Mamaroneck Avenue
  White Plains, NY 10605
  Phone: (888) 663-4637
  Fax: (914) 997-4763
  resourcecenter@modimes.org
  http://www.modimes.org

The March of Dimes is a national research and education foundation dedicated to the prevention of birth defects. The Resource Center staff of trained professionals provides information and referral services to the public. Staff members help people address personal and complex problems relating to numerous topics, including specific birth defects or infant health problems, pre-pregnancy, pregnancy, teen pregnancy, newborn care, drugs and environmental hazards during pregnancy, support groups, and genetics.

## MARCUS Developmental Institute

Division of Psychological Services
  1605 Chantilly Drive
  Atlanta, GA 30324
  Phone: (404) 727-9450
  Fax: (404) 727-9598
  ccoles@emory.edu
  http://www.marcus.org/

The Marcus Institute provides diagnostic and evaluation services and support programs to people with developmental disabilities, their families, and those who live and work with them.

## Motherisk

The Hospital for Sick Children
  555 University Avenue
  Toronto, Ontario
  Canada
  M5G 1X8
  Phone: (877) FAS-INFO ([877] 327-4636)
  http://www.motherisk.org

The Motherisk Program at The Hospital for Sick Children in Toronto is a clinical, research, and educational program dedicated to antenatal drug, chemical, and disease risk counseling. It is affiliated with the University of Toronto. Motherisk provides information and guidance about the safety or risk to the developing fetus or infant of maternal exposure to drugs, chemicals, diseases, radiation, and environmental agents. Motherisk counseling and research gives women who are pregnant, planning to become pregnant, or breast feeding, rational treatment and the means for making well-informed choices to ensure a healthy start for their children. The Alcohol and Substance Use Helpline offers information and counseling to pregnant and breast feeding women, their families, and health care providers.

## National Center on Birth Defects and Developmental Disabilities (NCBDDD)

Fetal Alcohol Syndrome Prevention Section
  Centers for Disease Control and Prevention
  4770 Buford Highway N.E., MS F-49
  Atlanta, GA 30341-3724
  Phone: (770) 488-7370
  Fax: (770) 488-7361
  http://www.cdc.gov/ncbddd/fas

The NCBDDD at the Centers for Disease Control and Prevention (CDC) seeks to promote optimal fetal, infant, and child development; prevent birth defects and childhood developmental disabilities; and enhance the quality of life and prevent secondary conditions among children, adolescents, and adults who are living with a disability. The fetal alcohol syndrome prevention office of the NCBDDD conducts FAS surveillance and prevention research.

## National Clearinghouse For Alcohol And Drug Information (NCADI)

P. O. Box 2345
  Rockville, MD 20847-2345
  Phone: (800) 729-6686
  http://www.health.org

NCADI is the information service of the Center for Substance Abuse Prevention of the Substance Abuse and Mental Health Services Administration in the U.S. Department of Health and Human Services. NCADI distributes information and materials concerning alcohol, tobacco, and other drug abuse prevention and treatment.

## National Council on Alcoholism and Drug Dependence, Inc. (NCADD)

20 Exchange Place
  Suite 2902
  New York, NY 10005
  Phone: (800) NCA-CALL
  national@ncadd.org
  http://www.ncadd.org

NCADD provides education and information regarding substance abuse and dependence. It advocates prevention, intervention, and treatment through offices in New York and Washington, and includes a nationwide network of affiliates that provide information on local treatment resources.

## National Information Center for Children and Youth with Disabilities (NICHCY)

P.O. Box 1492
  Washington, DC 20013-1492
  Phone: (800) 695-0285, (202) 884-8200
  Fax: (202) 884-8441
  http://www.nichcy.org
  nichcy@aed.org

NICHCY is a national information and referral center that provides information on disabilities and disability-related issues for families, educators, and other professionals. The center focuses on children and youth (birth to age 22). NICHCY’s services include personal responses to specific questions; NICHCY publications, including fact sheets on specific disabilities, state resource sheets, parent guides, bibliographies, and issue papers; referrals to other organizations and sources of help; and information searches of NICHCY’s databases and library.

## National Institute on Alcohol Abuse and Alcoholism (NIAAA)

6000 Executive Boulevard, Suite 409
  Bethesda, MD 20892-7003
  Phone: (301) 443-3860
  http://www.niaaa.nih.gov

NIAAA supports and conducts biomedical and behavioral research on the causes, consequences, treatment, and prevention of alcoholism and alcohol-related problems and disseminates information on all aspects of alcoholism. NIAAA hosts the Interagency Coordinating Committee on Fetal Alcohol Syndrome (ICCFAS). Members of the ICCFAS include representatives from multiple national agencies and organizations. The Committee’s objectives are to exchange information and to coordinate Federal strategies and programs in an effort to more effectively address alcohol-related birth defects on a national level.

## National Organization on Fetal Alcohol Syndrome (NOFAS)

216 G Street N.E.
  Washington, DC 20002
  Phone: (202) 785-4585
  Fax: (202) 466-6456
  information@nofas.org
  http://www.nofas.org

NOFAS is a nonprofit organization dedicated to eliminating birth defects caused by alcohol consumption during pregnancy and improving the quality of life for those individuals and families affected. NOFAS piloted many of its programs in Native American communities and takes a multicultural approach to prevention and healing. NOFAS includes the following program areas: national and community-based public awareness campaigns; a curriculum for medical and allied health students; training workshops and seminars for professional and lay audiences; youth outreach and peer education initiatives; and the NOFAS information, resource and referral clearinghouse.

## National Resource Center for Special Needs Adoptions

16250 Northland Drive, Suite 120
  Southfield, MI 48075
  Phone: (248) 443-7080
  Fax: (248) 443-7099
  sfc@Spaulding.org
  http://www.spaulding.org

This organization provides training, consultation, and informational materials on special-needs adoptions for professionals, organizations, and parents.

## Native American Women’s Health Education Resource Center

P.O. Box 572
  Lake Andes, SD 57356
  Phone: (605) 487-7072
  nativewoman@jgc.apc.org
  http://www.nativeshop.org/nawherc.html

The Resource Center was the first such center located on a reservation in the United States. It operates a fetal alcohol syndrome awareness program, along with many other programs to help people locally, nationally, and internationally. Other programs focus on domestic violence, AIDS prevention, and youth services.

## Substance Abuse and Mental Health Services Administration (SAMHSA)

5600 Fishers Lane
  Rockville, MD 20857
  Phone: (301) 443-8956
  Fax: (301) 443-9050
  info@samhsa.gov
  http://www.samhsa.gov

SAMHSA is the Federal agency charged with improving the quality and availability of prevention, treatment, and rehabilitative services in order to reduce illness, death, disability, and cost to society resulting from substance abuse and mental illnesses.

## Other FAS Internet Links

**FAS/FAE USA National Directory**
  http://www.mofas.org/fasdirect
**Fetal Alcohol Syndrome Community Resource Center**
  http://www.come-over.to/FASCRC
**Arium:** a nonprofit organization for the prevention of fetal alcohol syndrome.
  http://arium.org
**FASlink:** an on-line support/information source.
  http://www.acbr.com/fas/faslink.htm
**Fasstar.com:** Fetal Alcohol Syndrome Support, Training Advocacy and Resources.

